# 

**Published:** 1861-07

**Authors:** 


### CONTENTS.

### ORIGINAL COMMUNICATIONS.

PAGE.

ARTICLE I.—Gelseminum Sempervirens, as a remedy in Gonorrhœa. By T. A. Means, M. D., Memphis Tennesse., .......... 627

ARTICLE II.—Useful Recipes. Selected by John R. Cushing, M. D. D., South Butler, Alabama., .......... 635

### SELECTIONS.

Diabetes Melitus .......... 643
Clinical Lecture in Mercy Hospital .......... 652
Directions to Army Suageons .......... 657
On the Treatment of Uterine Inflammation .......... 663
Case of Congestion and Softening ot the Brain .......... 673
Scieence Aiding Justice .......... 680
The Cause of Milk Sickness .......... 682
A Fruitful Source of Epilepsy .......... 685

### EDITORIAL AND MISCELLANEOUS.

Typhoid Fever and Epidemic Dysentery .......... 686
Yellow Jasmine .......... 687
Recipes .......... 688
Our Journal and the War .......... 688

